# Contacts in general practice during the COVID-19 pandemic: a register-based study

**DOI:** 10.3399/BJGP.2021.0703

**Published:** 2022-10-18

**Authors:** Linda Huibers, Bodil Hammer Bech, Ulrik Bak Kirk, Per Kallestrup, Claus Høstrup Vestergaard, Morten Bondo Christensen

**Affiliations:** Research Unit for General Practice, Aarhus University, Aarhus.; Department of Public Health, Aarhus University, Aarhus.; Research Unit for General Practice, Aarhus University, Aarhus.; Research Unit for General Practice and Department of Public Health, Aarhus University, Aarhus.; Research Unit for General Practice, Aarhus University, Aarhus.; Research Unit for General Practice and Department of Public Health, Aarhus University, Aarhus.

**Keywords:** COVID-19, general practice, health equity, primary health care, telemedicine

## Abstract

**Background:**

The COVID-19 pandemic has altered the provision of health care and expanded telehealth consultations.

**Aim:**

To study the effect of the COVID-19 pandemic on contact patterns in general practice, and to identify patient groups at risk of losing care.

**Design and setting:**

Register-based study of Danish general practice, including daytime and out-of-hours (OOH) services.

**Method:**

All individuals residing in Denmark from 1 January 2017 to 31 October 2020 were included. The incidence rate for six contact types in general practice and adjusted incidence rate ratio were calculated by comparing the incidence rate in the pandemic period with the adjusted expected incidence rate based on the incidence rate in the pre-pandemic period.

**Results:**

The number of face-to-face in-clinic consultations declined during the lockdown in March 2020. A subsequent increase in the number of clinic consultations was observed, rising to a level above that of the pre-pandemic period; this increase resulted mainly from the introduction of telehealth consultations (that is, video and extended telephone). The number of daytime email consultations increased, whereas the number of daytime home visits decreased. Likewise, the number of OOH telephone consultations increased, whereas the number of OOH home visits and clinic consultations decreased. Consultation rates of patients who are vulnerable, that is, those with low education, old age, and comorbidity, were most adversely affected by the pandemic. The most adverse impact in OOH clinic consultations was seen for children aged 0–9 years.

**Conclusion:**

New methods are called for to ensure access to general practice for patients who are vulnerable during a pandemic. The potential of telehealth consultations should be further investigated.

## INTRODUCTION

The COVID-19 pandemic has altered the provision of health care. Across the world, telehealth consultations have widely replaced in-clinic consultations because of the risk of spreading severe acute respiratory syndrome coronavirus 2.^1^^–^4 Telehealth solutions can be defined as *‘remote delivery of healthcare services using information and communication technology’*.^5^ They include video consultation, telephone consultation, text/instant messaging, email consultation, and online patient portals.^6^

Several studies have reported lower use of primary and ambulatory care, and rapid increases in the use of remote consultations during the early phases of the COVID-19 pandemic.^7^^–^10 The pandemic and the introduction of virtual care might have caused variations in healthcare use in different populations,^9^^,^^10^ as video consultation may constitute a barrier to receiving health care for some patients.^8^ Thus, the shift to remote consultations may have exacerbated disparities in access to health care.^11^^–^13 The decline in contacts seemed less pronounced among females, older adults, patients with poor mental health, and patients with high expected healthcare use.^9^^,^^10^ The greatest decline was seen among parents making contact regarding children and among those with low expected healthcare use.^10^ Some delayed care occurred for health problems that could be postponed without harm, but some patients may have faced complications because of delayed treatment of acute medical issues or insufficient management of chronic illness.^14^ Even though these studies have added relevant knowledge, most have focused on regional daytime care.

The national registers in Denmark make it possible to study the entire population both during the day and outside office hours. More insight into the implications of COVID-19 and the introduction of virtual care is needed to optimise future healthcare provision. Moreover, there is a need to gain more knowledge about how to use these new telehealth opportunities in the best way in general practice. This study set out to explore the effect of the COVID-19 pandemic on contact patterns in general practice in Denmark and to identify patient groups at risk of reducing contacts with general practice.

## METHOD

### Design and population

A register-based time series study was conducted including all Danish residents from 1 January 2017 to 31 October 2020. The number of contacts with general practice during 2017–2019 was compared with the number of contacts during the first months of the COVID-19 pandemic.

**Table:** How this fits in

The COVID-19 pandemic has altered the provision of health care worldwide, and telehealth consultations have to some extent replaced traditional face-to-face in-clinic consultations to limit the risk of infection. This study found that a substantial part of in-clinic consultations were substituted with a new type of extended telephone consultation in daytime general practice. Video consultations replaced a considerable part of in-clinic consultations in out-of-hours services, but only a small part of in-clinic consultations in daytime general practice. Consultation rates of the most vulnerable patient groups were most adversely affected by the pandemic; this was seen for most contact types. The potential of telehealth consultations should be further investigated.

### Setting

In Denmark, general practice is tax-funded and free of charge for the patient. During the day, GPs provide care to their listed patients. GPs are remunerated through a fee per capita, but the main income (approximately 70%–75%) is based on fee-for-service reimbursement.

During the day (8.00 am to 4.00 pm), GPs offer a range of basic services, including face-to-face in-clinic consultations (regular, prenatal appointments, preventive child care, and conversational therapy for mental health issues), home visits, regular telephone consultations, and email consultations. Outside office hours, GPs are paid on a fee-for-service basis. In four of the five Danish regions, GPs run the out-of-hours (OOH) general practice service, also referred to as a GP cooperative, which patients must call to schedule an appointment. At the GP cooperative, GPs perform telephone triage and decide whether to offer a regular telephone consultation, refer for a face-to-face GP consultation (in the clinic or a home visit), or refer to hospital or emergency medical services. The OOH general practice service is open on weekdays between 4.00 pm and 8.00 am as well as at weekends and during holidays. Only the Capital Region of Denmark operates a different OOH healthcare service; the medical helpline 1813 (MH-1813). As data from MH-1813 are not available in the national registers, the Capital Region of Denmark was excluded from the current analyses about the use of general practice outside office hours but included in the analyses for daytime care.

Video consultation was rapidly introduced during the COVID-19 pandemic. To enhance the use of virtual care as an alternative to regular face-to-face in-clinic consultations in the daytime, the GPs could choose to perform a range of basic services by video or as an extended telephone consultation for health problems that would usually have prompted a face-to-face in-clinic consultation in the pre-pandemic period. Examples include consultations for prenatal appointments and preventive child care. However, an extended telephone consultation could not be used for conversational therapy. In OOH care, the GPs could use video consultations. For these consultations, new (temporary) remuneration codes were introduced, and these temporary codes could be used in combination with existing codes for reimbursement purposes.

### Outcome measures

The following outcome measure was defined: number of contacts with general practice per patient year (daytime, OOH, and all; stratified by basic remuneration codes before and during the pandemic). Contact types are registered with a range of remuneration codes. In this study, the term ‘virtual care’ refers to both video consultations and extended telephone consultations (see overview of remuneration codes in Supplementary Table S1). Preventive care contacts consisted of prenatal appointments and preventive child care. Furthermore, as extended telephone consultations and video consultations were new alternatives to contacts concerning health problems that were previously managed by face- to-face in-clinic consultations, in this study these were considered equivalent to face-to-face in-clinic consultations. Thus, clinic consultations consisted of three subtypes:

- regular face-to-face in-clinic consultations;
- video consultations; and
- extended telephone consultations.

The term ‘telephone consultations’ covered solely regular telephone consultations. The ‘extended telephone consultation’ was primarily for patients who were technically unable to participate in a video consultation. The word ‘extended’ refers to the fact that this consultation concerned more extended topics than normally handled by telephone consultations. Therefore, these extended telephone consultations were most often lengthier than regular telephone consultations. Email consultations are not part of clinic consultations but a separate contact type.

### Data collection

Data were collected from a range of national registers for the study period and these data were linked through the personal identification number. The National Health Insurance Service Register^15^ provided information on date, time, and type of contact with daytime GP and OOH general practice, and the services delivered (through remuneration codes). The National Patient Register holds records on hospital contacts (somatic, psychiatric, as well as private hospitals), and provided the diagnosis codes included in the Charlson Comorbidity Index.^16^ The Civil Registration System^17^ and Statistics Denmark delivered data on patient characteristics (age, sex, cohabitation, education, ethnicity, income, urbanisation, and employment status). Comorbidity was defined as the number of diagnoses included in the Charlson Comorbidity Index. Apart from age, sex, and comorbidity, all covariates were at a household level, for example, a household’s level of income was determined by its highest earning occupant. Thus, it was possible to avoid excluding contacts for children because of missing values. Furthermore, it was anticipated that socioeconomic characteristics (for example, education or income) at the household level would be stronger predictors for help-seeking behaviour than those at individual levels. Help-seeking is often discussed with, or suggested by, other members of the household,^18^ in particular for children, who have low levels of education and income in the registers. Prior to any analysis, individuals with missing values for income, employment status, or cohabitation were excluded, as this information was often missing concurrently and thus led to convergence issues for the model. This meant excluding 40 246 unique individuals (0.66%). For the remaining individuals, missing covariates were placed in a separate category.

### Analyses

The study population was followed from birth, immigration, or 1 January 2017 (whichever came last) until death, emigration, or 31 October 2020 (whichever came first). For each person, this period was divided into shorter time spans according to changes in covariates (see Supplementary Table S2). For each time span, the number of outcomes per resident was recorded along with the duration of each time span. However, age and sex of each resident were recorded at the beginning of each time span. Next, the number of outcomes and the durations were summed by month and year. Dividing the number of outcomes by the risk time provided the unadjusted observed incidence rate, which was plotted in categories of related remuneration codes. The date of 11 March 2020 was used as the starting date for the pandemic period, when the first official lockdown in Denmark was announced.^19^

To provide adjusted incidence rate ratios (IRRs), Poisson regressions were run for each group of remuneration codes on the data for 2017–2019 (that is, the pre- pandemic period), with risk time serving as the offset, and adjusted for the following covariates: sex, age, cohabitation, education, ethnicity, comorbidity, income, urbanisation, employment status, month, and month-ID (ID number of month in dataset), with the latter being treated as a continuous linear effect. Seasonality was taken into account through adjustment for month. This made it possible to calculate the expected utilisation (expected incidence rate) of general practice throughout the pandemic period as an extrapolation of previous help-seeking. Dividing the observed incidence rate by the adjusted expected incidence rate gave the adjusted IRR, which was plotted as curves according to groups of related remuneration codes. The change because of the pandemic was calculated by subtracting the expected number of contacts from the observed number of contacts after 11 March 2020 and the results (that is, overall effect) are presented as a percentage of the expected number of consultations.

Finally, to see if changes in contact patterns were evenly distributed within subsets of the population, modifications of the pandemic effect within each of the covariates were looked for. This was done by using fully adjusted Poisson models, one for each covariate, and each with an interaction term for the covariate in question. Results were presented in a forest plot. Stata (version 16) was used for all analyses.

## RESULTS

Contact patterns varied between the pre-pandemic period and the pandemic period, and variations in adjusted numbers were seen for both daytime and OOH general practice (Table 1).

**Table 1.** Unadjusted number of contacts with general practice (daytime and OOH) in the pre-pandemic and pandemic period

**Characteristic**	**Pre-pandemic, contacts, *n* per person–year**	**Pandemic, contacts, *n* per person–year**	**Difference**	**Change in total number of contacts (in tens of thousands)**	**RIR^a^**
**Daytime**					
Clinic consultations^b^	3.25	3.13	−0.12	−44.69	−0.04
Regular in-clinic	3.25	2.13	−1.12	−416.41	−0.35
Extended telephone	0.0	0.95	0.95	352.76	—
Video	0.0	0.05	0.05	18.96	—
Regular telephone consultations	1.73	1.51	−0.22	−81.29	−0.13
Home visits	0.08	0.09	0.01	3.81	0.13
Email consultations	1.24	1.65	0.41	151.53	0.33
Preventive care consultations^c^	0.07	0.07	0.0	−0.76	−0.03
Regular in-clinic	0.07	0.06	0.0	−0.97	−0.04
Extended telephone	0.0	0.0	0.0	0.16	—
Video	0.0	0.0	0.0	0.04	—
Conversational therapy	0.06	0.05	−0.01	−2.85	−0.13
Regular in-clinic	0.06	0.04	−0.02	−5.87	−0.26
Extended telephone	0.0	0.0	0.0	0.44	—
Video	0.0	0.01	0.01	2.58	—

**OOH**					
Clinic consultations	0.15	0.11	−0.05	−13.12	−0.3
Regular in-clinic	0.15	0.08	−0.07	−19.88	−0.45
Video	0.0	0.02	0.02	6.76	—
Regular telephone consultations	0.26	0.31	0.05	15.34	0.21
Home visits	0.04	0.03	−0.01	−3.91	−0.35

a *As video consultations and extended telephone consultations were first introduced at the start of the COVID-19 pandemic, the model could not calculate the RIR.*

b *During the pandemic, clinic consultations could be provided as regular in-clinic consultations, extended telephone consultations (separate remuneration code), or video consultations (separate remuneration code).*

c *Preventive care consultations include prenatal appointments and preventive child care. OOH = out of hours. RIR = relative incidence rate reduction.*

**Figure 1.**
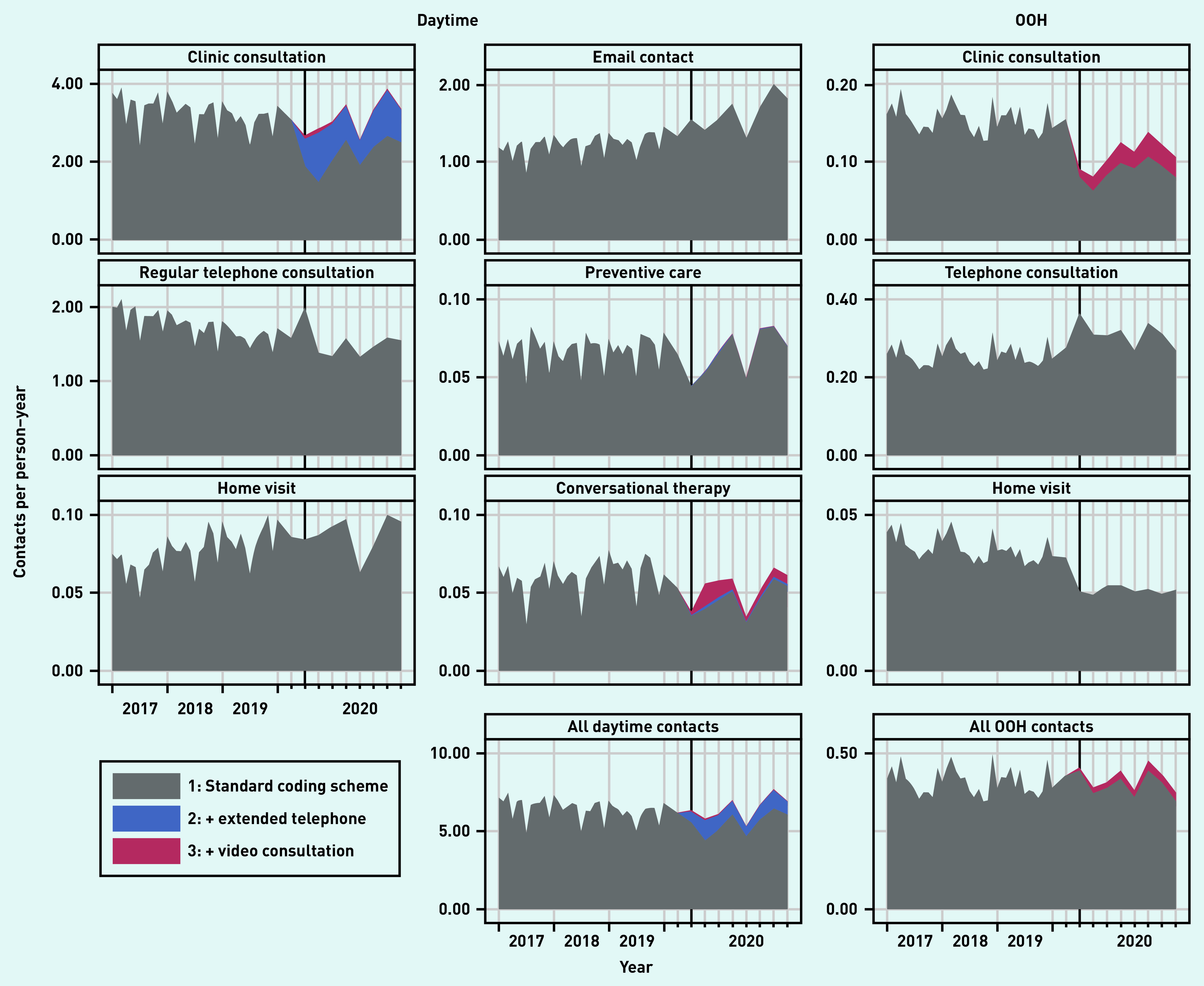
*Number of remuneration codes used per person–year from 1 January 2017 to 31 October 2020. Remuneration codes were added stepwise for the pandemic period: 1) regular remuneration codes; 2) plus extended telephone consultation (daytime only); and 3) plus video consultations. For readability, 2020 was the focus. Clinic consultations included regular in-clinic consultations, extended telephone consultations, and video consultations. OOH = out of hours.*

**Figure 2.**
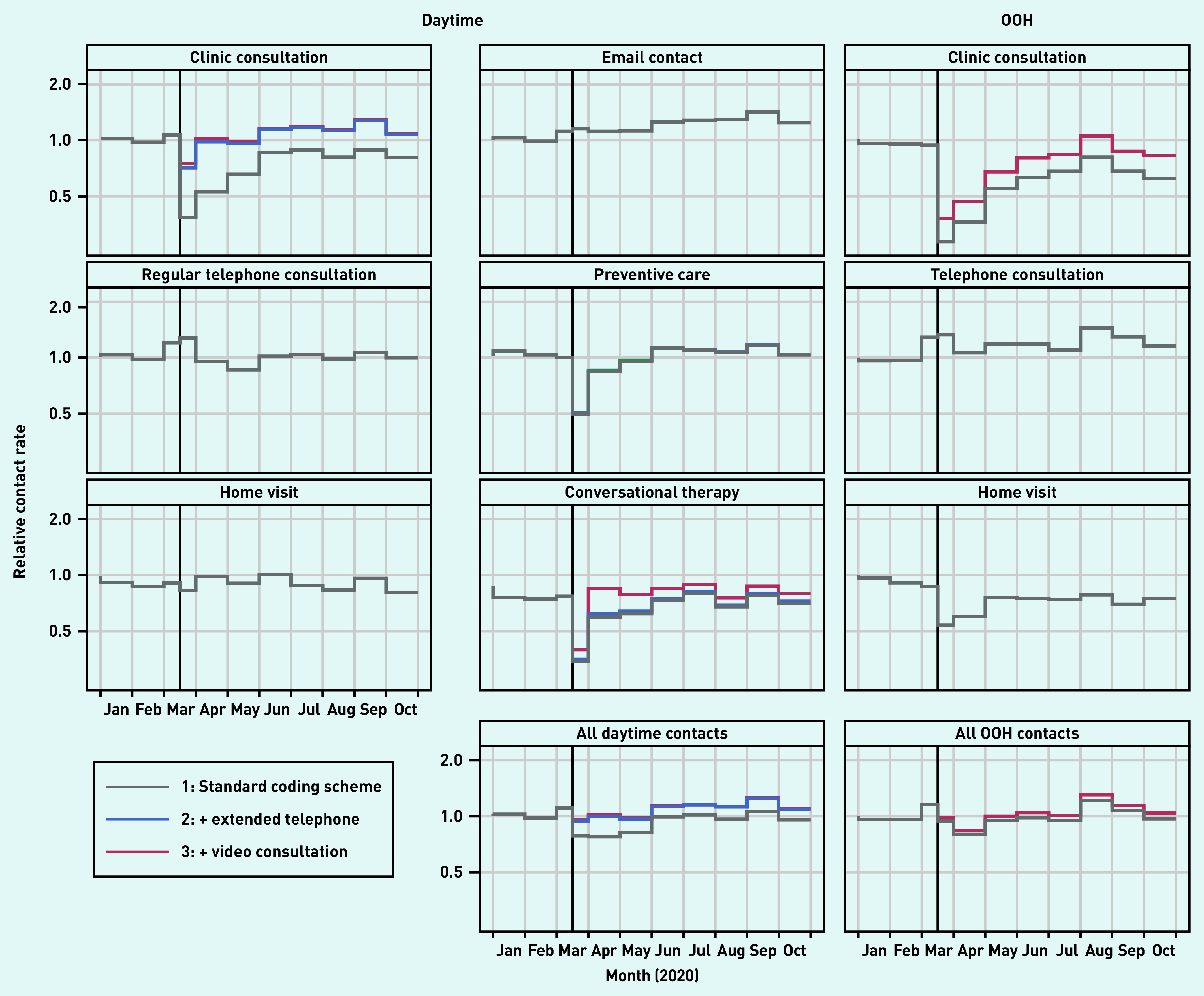
*Relative number of contacts (observed contacts/expected contacts) based on a prediction model that includes patient characteristics and a linear time trend based on 2017–2019 data. Remuneration codes are added stepwise for the pandemic period: 1) regular remuneration codes; 2) plus extended telephone consultation (daytime only); and 3) plus video consultations. Clinic consultations included regular in-clinic consultations, extended telephone consultations, and video consultations. OOH = out of hours.*

Figure 1 presents the total number of contacts for the basic services. A population description is presented in Supplementary Table S3. Figure 2 presents the contact rate relative to the rate predicted by the model during the pandemic (presenting the same overall picture as seen in Figure 1). The clinic consultations showed an initial drop of 25% in March 2020, but this number increased to above pre-pandemic levels soon thereafter (IRRs ranging from 0.98 to 1.29); this was mainly as a result of extended telephone consultations (proportion ranging from 27% to 46% of all clinic contacts) and video consultations (ranging from 1% to 4%) (Figure 2).

A similar pattern was seen for preventive care consultations. Regular telephone consultations peaked at the start of the pandemic (IRR 1.28 in March), dropped for a few months, and ended around pre-pandemic levels (IRRs ranging from 0.99 to 1.07). Home visits remained mostly below the pre-pandemic levels (IRRs ranging from 0.81 to 1.01), as did conversational therapy. Email consultations significantly increased during the pandemic (IRRs ranging from 1.12 to 1.42) (Figure 2).

At the OOH general practice services, clinic consultations dropped considerably in the first 3 months (IRRs ranging from 0.38 to 0.68). Thereafter, the number of OOH clinic consultations remained mostly below pre-pandemic levels (IRRs ranging from 0.62 to 1.06), even though video consultations were used in up to 24% of all clinic consultations. The number of home visits kept below that of the pre-pandemic level (IRRs ranging from 0.54 to 0.79), whereas the number of regular telephone consultations stayed above (IRRs ranging from 1.07 to 1.45) (Figure 2).

As seen in Table 2, daytime GP contacts increased by 9.9% in the pandemic period, relative to what was to be expected. This increase was driven primarily by clinic consultations (8.6%) and email consultations (24.2%). Contact with OOH primary care increased by 4.3%, relative to what was to be expected, which was mainly because of regular telephone consultations (21.5%), with large decreases in clinic consultations (−25.9%) and home visits (−29.4%).

**Table 2.** Adjusted overall effect of the pandemic on contacts with general practice (daytime and OOH)

**Characteristic**	**Predicted contacts,^a^ *n* (in tens of thousands)**	**Observed contacts, *n* (in tens of thousands)**	**Difference, *n* (in tens of thousands)**	**Overall effect, %**
**Daytime**	2187.6	2404.4	216.8	9.9
Clinic consultations^b^	1068.5	1160.4	92.0	8.6
Regular in-clinic	1068.5	788.7	−279.7	−26.2
Extended telephone	0.0	352.8	352.8	NA^c^
Video	0.0	19.0	19.0	NA^c^
Regular telephone consultations	550.8	560.7	9.9	1.8
Home visits	35.8	32.4	−3.4	−9.6
Email consultations	491.4	610.4	119.0	24.2
Preventive care consultations^b^	24.2	24.3	0.1	0.4
Regular in-clinic	24.2	24.1	−0.1	−0.4
Extended telephone	0.0	0.2	0.2	NA^c^
Video	0.0	0.0	0.0	NA^c^
Conversational therapy^b^	24.9	19.5	−5.4	−21.6
Regular in-clinic	24.9	16.5	−8.4	−33.7
Extended telephone	0.0	0.4	0.4	NA^c^
Video	0.0	2.6	2.6	NA^c^

**OOH**	115.2	120.2	5.0	4.3
Clinic consultations	41.9	31.1	−10.9	−25.9
Regular in-clinic	41.9	24.3	−17.6	−42.0
Video	0.0	6.8	6.8	NA^c^
Regular telephone consultations	73.4	89.1	15.8	21.5
Home visits	10.4	7.3	−3.0	−29.4

a *Prediction based on data from 2017 to 2019, adjusted for patient characteristics, month, and year.*

b *Includes regular face-to-face in-clinic consultations, extended telephone consultations, and video consultations.*

c *As video consultations and extended telephone consultations were first introduced at the start of the COVID-19 pandemic, the model could not predict such outcomes. Because of rounding estimates data in some rows may not appear to add up to 100%. NA = not applicable. OOH = out of hours.*

**Figure 3.**
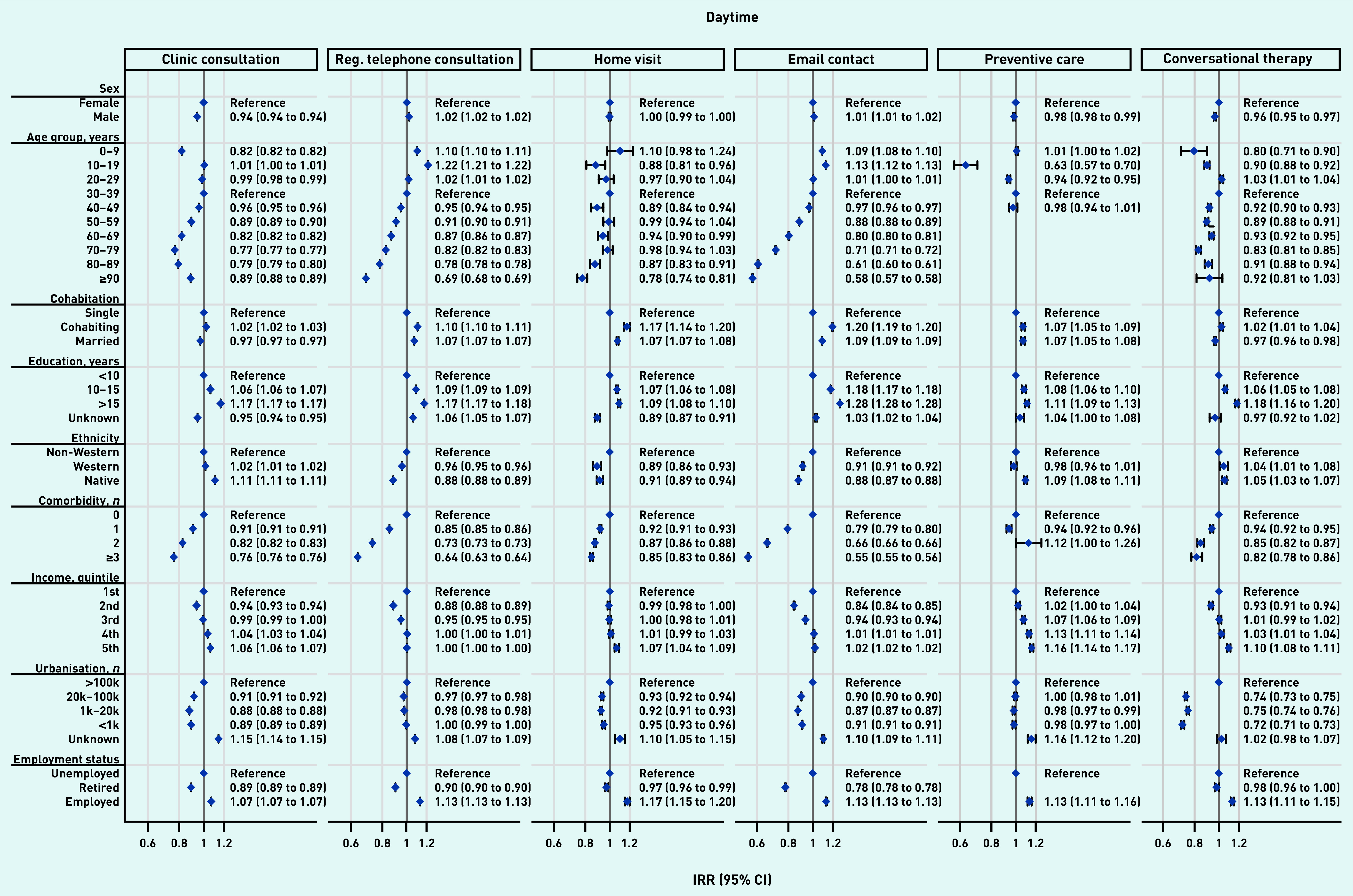
*Adjusted IRRs for the impact of the pandemic on daytime general practice contacts during the pandemic compared with before the pandemic for patient characteristics, stratified by basic type of contacts. In the daytime, the number of clinic consultations increased by 8.6%, regular telephone consultations increased by 1.8%, home visits decreased by 9.6%, email consultations increased by 24.2%, preventive care consultations increased by 0.4%, and conversational therapy decreased by 21.6% (see Table 2).All percentages are adjusted for patient characteristics, month, and year (see Table 2). For example, patients aged ≥40 years experienced a smaller increase in daytime clinic consultations compared with the pre-pandemic period than those aged 30–39 years. Clinic consultations included regular in-clinic consultations, extended telephone consultations, and video consultations. Comorbidity is the number of diagnoses included in the Charlson Comorbidity Index. IRR = incidence rate ratio. Reg. = regular.*

**Figure 4.**
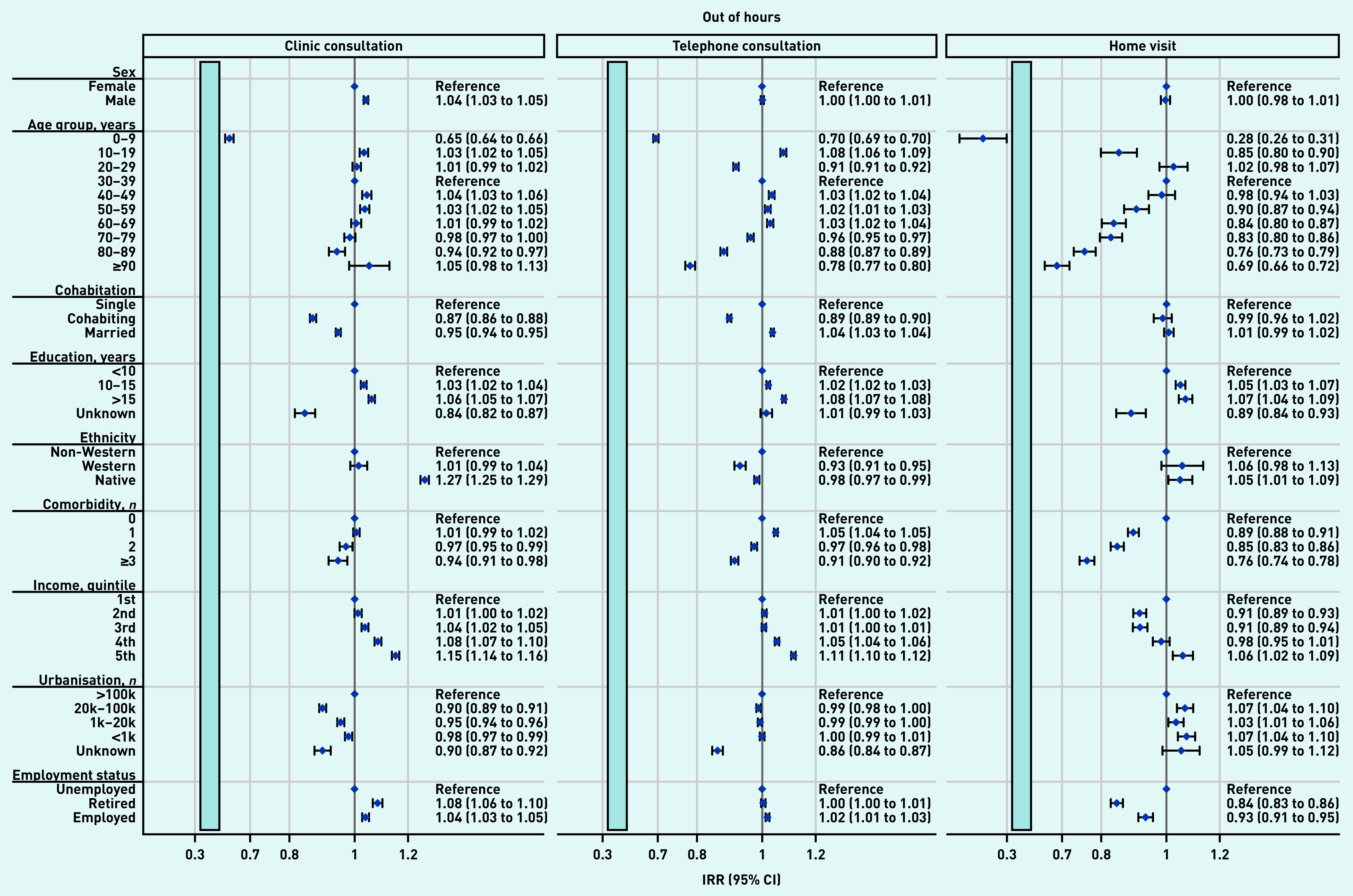
*Adjusted IRRs for the impact on OOH general practice contacts during the pandemic compared with before the pandemic for patient characteristics, stratified by type of contact. OOH, the number of clinic consultations decreased by 25.9%, regular telephone consultations increased by 21.5%, and home visits decreased by 29.4% (see Table 2). All percentages were adjusted for patient characteristics, month, and year (see Table 2). For example, patients aged 0–9 years experienced a larger decrease in clinic consultations with OOH services compared with the pre-pandemic period than those aged 30–39 years. Clinic consultations included regular in-clinic consultations, extended telephone consultations, and video consultations. Comorbidity is the number of diagnoses included in the Charlson Comorbidity Index. The light blue bars illustrate that the x-axis is interrupted to aid readability. IRR = incidence rate ratio. OOH = out of hours. Reg. = regular.*

The overall effects shown in Table 2 were not distributed equally across patient groups. Figures 3 and 4 present the impact of the pandemic on daytime general practice and OOH contacts, respectively, during the pandemic compared with before the pandemic for patient groups. Across all types of contacts, consultation rates of vulnerable patients (that is, those being older, being unemployed/retired, with a lower educational level, lower income level, and experiencing existing comorbidity) were more adversely affected by the pandemic than more affluent patients. Compared with older patients, children aged 0–9 years experienced the largest adverse impact in daytime contacts (here together with patients aged 60–89 years) and in OOH contacts. Patients from suburban and rural areas (population ≤100 000) also experienced a larger adverse impact than patients from urban areas (population >100 000).

## DISCUSSION

### Summary

At the start of lockdown in March 2020, the number of clinic consultations declined steeply. This was quickly followed by a countertrend towards and even above pre-pandemic levels, which was prompted mainly by the introduction of extended telephone consultations and video consultations (this trend was most distinct for OOH services). In general, the largest decrease in contacts was seen for the patients who were most vulnerable.

### Strengths and limitations

A large dataset was used in the current study, including all general practice contacts in Denmark and various patient characteristics. The results of this study are generalisable to other countries with a similar setting using GP gatekeeping and that are free of charge for the patient. Data based on regular coding are useful for research purposes, but some reservations may exist about their validity.^15^ The economic incentive to register services contributes to completeness, in particular for regular remuneration codes. The reliability of the GPs’ use of the hastily implemented COVID-19 remuneration codes is unknown, and the GPs might have had varying practices. Therefore, possible misclassification of contact types cannot be ruled out. In the current study whether the contact rate was lower for certain patient groups during the pandemic compared with the pre-pandemic period was explored, but the study design did not allow the authors to assess whether this was because of a lower level of illness, reluctance to contact (because of fear of infection or overburdening the health services), or reduced accessibility and availability of general practice. Hospital-based data were used to calculate comorbidity, using the list of diagnoses from the Charlson Comorbidity Index. This may have led to underestimation of comorbidity^20^ as this list is limited and as patients with mild chronic diseases are often treated solely in general practice.

### Comparison with existing literature

Several other studies have also reported lower use of general practice^9^^,^^10^^,^^14^ and rapid increases in virtual care during the early phases of the COVID-19 pandemic.^7^^,^^10^^,^^14^^,^^21^^–^23 The reported decrease varies from 16% to 79% for in-clinic consultations in the daytime,^7^^,^^9^^,^^10^^,^^14^^,^^24^ and in the current study a monthly decrease of up to 25% for daytime clinic consultations and up to 62% for OOH clinic consultations was found. The share of virtual care (video and telephone) has varied considerably between studies, ranging from 19% to 90% of all consultations^7^^,^^9^^–^11^,^^14^^,^^21^ whereas the current study showed that up to 31% of all daytime consultations and up to 18% of all OOH clinic consultations were conducted as virtual care. However, in the current study when regular telephone consultations were added to video consultations and extended telephone consultations, this percentage increased to 53% of all daytime contacts and 78% of all OOH contacts. Several studies also found a countertrend in the total number of visits, which led to an average of near-pre-pandemic levels.^14^^,^^25^

The largest relative decrease in contacts in the current study was seen among patients who were vulnerable, but a Canadian study found that the patient groups with the highest care needs, including older patients and patients with high morbidity levels, maintained high levels of care during the course of the pandemic.^10^ Likewise, British GPs and nurses have been shown to keep a focus on patients who are vulnerable.^9^ Most governments and public authorities encouraged the population to limit contacts with healthcare services and change their help-seeking behaviour. Anxiety in the population about contracting COVID-19 at a health centre may have contributed to the decrease in contacts.^13^ Patients who are vulnerable may have had even more restrictive behaviour compared with other patient groups. The pandemic resulted in postponement of most chronic disease monitoring, health checks, preventive care, and screening activity, as these were not deemed ‘essential’.^14^^,^^26^ Additionally, the shift towards virtual care may have altered the contact patterns, in particular for older patients and patients with multiple chronic health problems.^8^^,^^12^^,^^13^^,^^27^^,^^28^

Finally, children aged 0–9 years experienced the most severe adverse impact on daytime clinic consultations and OOH contacts. Social isolation because of lockdown measures, such as the closing of schools and day care facilities, in combination with social distancing resulted in a decline in non-COVID-19 infectious diseases, in particular respiratory tract infections in children.^29^ Several studies found a prominent decrease in antibiotics prescribing for children aged 0–11 years during the COVID-19 pandemic.^30^^–^32 Furthermore, patients with respiratory tract infections were kept out of waiting rooms, which most likely affected children most.

### Implications for research and practice

Several studies have indicated concerns that the management of patients with chronic illness may now be lagging behind because of the pandemic.^13^^,^^14^^,^^26^ Some of the lost contacts are likely to be related to medically unnecessary or non-urgent short-term health problems, as well as to a lower incidence of non-COVID-19 infections because of lockdown measures and social distancing, whereas others may have caused delayed diagnosis and treatment of medical problems or delayed management of chronic illness.^10^^,^^14^ As this might have led to increased morbidity and mortality unrelated to COVID-19,^33^ future research should address the (long-term) effects of the pandemic on vulnerable patient groups. Furthermore, ways to support vulnerable patient groups in the use of virtual care technology should be investigated.

## Data

The data are not publicly available. All data analysed in the study were de-identified and stored on a secure server at Statistics Denmark. The datasets are available after formal application to Statistics Denmark.
